# Supplementary material for: Cell-type specific epigenetic clocks to quantify biological age at cell-type resolution
Source: Aging (Albany NY). 2024 Dec 29;16(22):13452–504. doi: 10.18632/aging.206184 (PMC11723652; doi:10.18632/aging.206184)
Supplement: Supplementary Methods [file aging-16-206184-s001.pdf]

SUPPLEMENTARY METHODS

DNA methylation datasets used in validation of the neuron/glia and brain-specific clocks

To validate all clocks and to demonstrate their specificity, we assembled a large collection of Illumina DNAm datasets, encompassing sorted brain cell (6 independent datasets) [1–6], bulk brain tissue (19 independent datasets) [2, 7–20] and other sorted non-brain cell-types and tissue-types. We restricted the assessment of the clocks to the control samples, to avoid potential confounding by disease. Below we provide details of the processing of the brain-related DNAm datasets used in the validation.

Sorted neuron and glia datasets

Pai

The EPIC dataset from Pai et al. [1] profiled DNAm in human frontal cortex sorted neuron from controls was obtained from the NCBI GEO website under the accession number GSE112179 (https://www.ncbi.nlm.nih.gov/geo/query/acc.cgi?acc=GSE112179). The file "GSE112179_RAW.tar" which contains the IDAT files was downloaded and processed with minfi package. Probes with NAs (defined by $P > 0.01$) were discarded. The filtered data was subsequently normalized with BMIQ, resulting in a normalized data matrix for 855735 probes and 100 samples.

Gasparoni

The 450k dataset from Gasparoni et al. [2] profiled DNAm in human occipital cortex sorted neuron and gliacyte from controls was obtained from the NCBI GEO website under the accession number GSE66351 (https://www.ncbi.nlm.nih.gov/geo/query/acc.cgi?acc=GSE66351). The file "GSE66351_RAW.tar" which contains the IDAT files was downloaded and processed with minfi package. Probes with NAs (defined by $P > 0.01$) were discarded. The filtered data was subsequently normalized with BMIQ, resulting in a normalized data matrix for 482176 probes and 62 samples.

Kozlenkov

The 450k dataset from Kozlenkov et al. [3] profiled DNAm in human frontal cortex sorted neuron from controls was obtained from the NCBI GEO website under the accession number GSE98203 (https://www.ncbi.nlm.nih.gov/geo/query/acc.cgi?acc=GSE98203). The file "GSE98203_RAW.tar" which contains the IDAT files was downloaded and processed with minfi package. Probes with NAs (defined by $P > 0.01$) were discarded. The filtered data was subsequently normalized with BMIQ, resulting in a normalized data matrix for 469902 probes and 88 samples.

Guintivano

The 450k dataset from Guintivano et al. [4] profiled DNAm in human frontal cortex sorted neuron and gliacyte from controls was obtained from the NCBI GEO website under the accession number GSE41826 (https://www.ncbi.nlm.nih.gov/geo/query/acc.cgi?acc=GSE41826). The file "GSE41826_RAW.tar" which contains the IDAT files was downloaded and processed with minfi package. Probes with NAs (defined by $P > 0.01$) were discarded. The filtered data was subsequently normalized with BMIQ, resulting in a normalized data matrix for 482469 probes and 58 samples.

Hannon

The EPIC dataset from Hannon et al. [5] profiled DNAm in human frontal cortex sorted neuron and gliacyte from controls was obtained from the NCBI GEO website under the accession number GSE234520 (https://www.ncbi.nlm.nih.gov/geo/query/acc.cgi?acc=GSE234520). The file "GSE234520_RAW.tar" which contains the IDAT files was downloaded and processed with minfi package. Probes with NAs (defined by $P > 0.01$) were discarded. The filtered data was subsequently normalized with BMIQ, resulting in a normalized data matrix for 812778 probes and 103 samples.

Witte

The EPIC dataset from Witte et al. [6] profiled DNAm in human four different brain regions, medial frontal gyrus (MFG), superior temporal gyrus (STG), subventricular zone (SVZ) and thalamus (THA) sorted gliacyte from controls was obtained from the NCBI GEO website under the accession number GSE191200 (https://www.ncbi.nlm.nih.gov/geo/query/acc.cgi?acc=GSE191200). The file "GSE191200_RAW.tar" which contains the IDAT files was downloaded and processed with minfi package. Probes with NAs (defined by $P > 0.01$) were discarded. The filtered data was subsequently normalized with BMIQ, resulting in a normalized data matrix for 838988 probes and 56 samples.

Brain bulk datasets

Murphy

The 450k dataset from Murphy et al. [7] profiled DNAm in human PFC from controls was obtained from the NCBI GEO website under the accession

number GSE88890 (https://www.ncbi.nlm.nih.gov/geo/query/acc.cgi?acc=GSE88890). The files "GSE88890_methylatedIntensities.csv.gz", "GSE88890_unmethylatedIntensities.csv.gz", "GSE88890_detectionP.csv.gz" were downloaded and processed with minfi package. Probes with > 3% NAs (defined by *P* > 0.01) were discarded. The filtered data was subsequently normalized with BMIQ, resulting in a normalized data matrix for 416054 probes and 75 samples.

Rydbirk

The EPIC dataset from Rydbirk et al. [8] profiled DNAm in human PFC from controls was obtained from the NCBI GEO website under the accession number GSE143157 (https://www.ncbi.nlm.nih.gov/geo/query/acc.cgi?acc=GSE143157). The file "GSE143157_RAW.tar" which contains the IDAT files was downloaded and processed with minfi package. Probes with NAs (defined by *P* > 0.01) were discarded. The filtered data was subsequently normalized with BMIQ, resulting in a normalized data matrix for 831166 probes and 78 samples.

Torabi

The 450K dataset from Torabi et al. [9] profiled DNAm in human PFC from controls was obtained from the NCBI GEO website under the accession number GSE128601 (https://www.ncbi.nlm.nih.gov/geo/query/acc.cgi?acc=GSE128601). The file "GSE128601_Matrix_signal_intensities_scz.txt.gz" was downloaded and processed with minfi package. Probes with NAs (defined by *P* > 0.01) were discarded. The filtered data was subsequently normalized with BMIQ, resulting in a normalized data matrix for 478722 probes and 125 samples.

Xu

The 450K dataset from Xu et al. [10] profiled DNAm in human PFC from controls was obtained from the NCBI GEO website under the accession number GSE49393 (https://www.ncbi.nlm.nih.gov/geo/query/acc.cgi?acc=GSE49393). The file "GSE49393_signal_intensities.txt.gz" was downloaded and processed with minfi package. Probes with NAs (defined by *P* > 0.01) were discarded. The filtered data was subsequently normalized with BMIQ, resulting in a normalized data matrix for 482931 probes and 48 samples.

Huynh

The 450K dataset from Huynh et al. [11] profiled DNAm in human frontal lobe from controls was obtained from the NCBI GEO website under the accession number GSE40360 (https://www.ncbi.nlm.nih.gov/geo/query/acc.cgi?acc=GSE40360). The file "GSE40360_RAW_nonorm_nobg.txt.gz" was downloaded and processed with minfi package. Probes with NAs (defined by *P* > 0.01) were discarded. The filtered data was subsequently normalized with BMIQ, resulting in a normalized data matrix for 474136 probes and 46 samples.

Viana2

The 450K dataset from Viana et al. [12] profiled DNAm in human hippocampus from controls was obtained from the NCBI GEO website under the accession number GSE89703 (https://www.ncbi.nlm.nih.gov/geo/query/acc.cgi?acc=GSE89703). The files "GSE89703_methylatedintensities_HC_LNDBB.csv.gz", "GSE89703_unmethylatedintensities_HC_LNDBB.csv.gz", "GSE89703_detectionP_HC_LNDBB.csv.gz" were downloaded and processed with minfi package. Probes with NAs (defined by *P* > 0.01) were discarded. The filtered data was subsequently normalized with BMIQ, resulting in a normalized data matrix for 413242 probes and 27 samples.

Viana3

The 450K dataset from Viana et al. [12] profiled DNAm in human striatum, putamen from controls was obtained from the NCBI GEO website under the accession number GSE89705 (https://www.ncbi.nlm.nih.gov/geo/query/acc.cgi?acc=GSE89705). The files "GSE89705_methylatedintensities_STR_DBCBB.csv.gz", "GSE89705_unmethylatedintensities_STR_DBCBB.csv.gz", "GSE89705_detectionP_STR_DBCBB.csv.gz" were downloaded and processed with minfi package. Probes with NAs (defined by *P* > 0.01) were discarded. The filtered data was subsequently normalized with BMIQ, resulting in a normalized data matrix for 413468 probes and 33 samples.

Viana4

The 450K dataset from Viana et al. [12] profiled DNAm in human striatum, putamen from controls was obtained from the NCBI GEO website under the accession number GSE89706 (https://www.ncbi.nlm.nih.gov/geo/query/acc.cgi?acc=GSE89706). The files "GSE89706_methylatedintensities_STR_LNDBB.csv.gz", "GSE89706_unmethylatedintensities_STR_LNDBB.csv.gz", "GSE89706_detectionP_STR_LNDBB.csv.gz" were downloaded and processed with minfi package. Probes with NAs (defined by *P* > 0.01) were discarded. The filtered data was subsequently normalized with BMIQ, resulting in a normalized data matrix for 418738 probes and 49 samples.

Markunas

The EPIC dataset from Markunas et al. [13] profiled DNAm in human nucleus accumbens from controls was obtained from the NCBI GEO website under the accession number GSE147040 (https://www.ncbi.nlm.nih.gov/geo/query/acc.cgi?acc=GSE147040). The file

"GSE147040_matrix_intensities_for_geo.txt.gz", was downloaded and processed with minfi package. Probes with NAs (defined by $P > 0.01$) were discarded. The filtered data was subsequently normalized with BMIQ, resulting in a normalized data matrix for 850934 probes and 221 samples.

Viana1

The 450K dataset from Viana et al. [12] profiled DNAm in human cerebellum from controls was obtained from the NCBI GEO website under the accession number GSE89702 (https://www.ncbi.nlm.nih.gov/geo/query/acc.cgi?acc=GSE89702). The files "GSE89702_methylatedintensities_CER_DBCBB.csv.gz", "GSE89702_unmethylatedintensities_CER_DBCBB.csv.gz", "GSE89702_detectionP_CER_DBCBB.csv.gz" were downloaded and processed with minfi package. Probes with NAs (defined by $P > 0.01$) were discarded. The filtered data was subsequently normalized with BMIQ, resulting in a normalized data matrix for 413811 probes and 33 samples.

Stefania1

The EPIC dataset from Stefania et al. [14] profiled DNAm in human cerebellum from controls was obtained from the NCBI GEO website under the accession number GSE137222 (https://www.ncbi.nlm.nih.gov/geo/query/acc.cgi?acc=GSE137222). The files "GSE137222_methylatedIntensities_AI.csv.gz", "GSE137222_unmethylatedIntensities_AI.csv.gz", "GSE137222_detectionP_AI.csv.gz" were downloaded and processed with minfi package. Probes with NAs (defined by $P > 0.01$) were discarded. The filtered data was subsequently normalized with BMIQ, resulting in a normalized data matrix for 794359 probes and 58 samples.

Stefania2

The 450k dataset from Stefania et al. [14] profiled DNAm in human cerebellum from controls was obtained from the NCBI GEO website under the accession number GSE137223 (https://www.ncbi.nlm.nih.gov/geo/query/acc.cgi?acc=GSE137223). The files "GSE137223_methylatedIntensities_ED.csv.gz", "GSE137223_unmethylatedIntensities_ED.csv.gz", "GSE137223_detectionP_ED.csv.gz" were downloaded and processed with minfi package. Probes with NAs (defined by $P > 0.01$) were discarded. The filtered data was subsequently normalized with BMIQ, resulting in a normalized data matrix for 427624 probes and 33 samples.

Semick

The 450k dataset from Semick et al. [15] profiled DNAm in human dorsolateral prefrontal cortex (DLPFC), hippocampus (HIPPO), entorhinal cortex (ERC), and cerebellum (CRB) from controls and AD cases was obtained from the NCBI GEO website under the accession number GSE125895 (https://www.ncbi.nlm.nih.gov/geo/query/acc.cgi?acc=GSE125895). The file "GSE125895_RAW.tar" which contains the IDAT files was downloaded and processed with minfi package. Probes with NAs (defined by $P > 0.01$) were discarded. The filtered data was subsequently normalized with BMIQ, resulting in a normalized data matrix for 437426 probes and 269 samples.

Pidsley

The 450k dataset from Pidsley et al. [16] profiled DNAm in human prefrontal cortex (PFC), entorhinal cortex (EC), superior temporal gyrus (STG) from controls and AD cases was obtained from the NCBI GEO website under the accession number GSE43414 (https://www.ncbi.nlm.nih.gov/geo/query/acc.cgi?acc=GSE43414). The file "GSE43414_signal_intensities.csv.gz" was downloaded and processed with minfi package. Probes with NAs (defined by $P > 0.01$) were discarded. The filtered data was subsequently normalized with BMIQ, resulting in a normalized data matrix for 474819 probes and 250 samples.

Smith

The 450k dataset from Smith et al. [17] profiled DNAm in human prefrontal cortex (PFC), superior temporal gyrus (STG) from controls and AD cases was obtained from the NCBI GEO website under the accession number GSE80970 (https://www.ncbi.nlm.nih.gov/geo/query/acc.cgi?acc=GSE80970). The files "GSE80970_MSACF_raw_methylated.txt.gz", "GSE80970_MSACF_raw_unmethylated.txt.gz", "GSE80970_MSACF_raw_pvals.txt.gz" were downloaded and processed with minfi package. Probes with NAs (defined by $P > 0.01$) were discarded. The filtered data was subsequently normalized with BMIQ, resulting in a normalized data matrix for 400811 probes and 286 samples.

Horvath

The 450k dataset from Horvath et al. [18] profiled DNAm in human frontal lobe (FL), temporal lobe (TL), parietal lobe (PL), motor cortex (MC), sensory cortex (SC), visual cortex (VC), cingulate gyrus (CG) from controls and AD cases was obtained from the NCBI GEO website under the accession number GSE72778 (https://www.ncbi.nlm.nih.gov/geo/query/acc.cgi?acc=GSE72778). The file "GSE72778_datSignal.csv.gz" was downloaded and processed with minfi package. Probes with NAs (defined by $P > 0.01$) were discarded. The filtered data was subsequently normalized with BMIQ, resulting in a normalized data matrix for 420402 probes and 300 samples.

Gasparoni

The 450k dataset from Gasparoni et al. [2] profiled DNAm in human frontal lobe (FL), temporal lobe (TL) from controls and AD cases was obtained from the NCBI GEO website under the accession number GSE66351 (https://www.ncbi.nlm.nih.gov/geo/query/acc.cgi?acc=GSE66351). The file "GSE66351_RAW.tar" which contains the IDAT files was downloaded and processed with minfi package. Probes with NAs (defined by $P > 0.01$) were discarded. The filtered data was subsequently normalized with BMIQ, resulting in a normalized data matrix for 474413 probes and 128 samples.

Watson

The 450k dataset from Watson et al. [19] profiled DNAm in superior temporal gyrus (STG) from controls and AD cases was obtained from the NCBI GEO website under the accession number GSE76105 (https://www.ncbi.nlm.nih.gov/geo/query/acc.cgi?acc=GSE76105). The file "GSE76105_Raw_Data.txt.gz" was downloaded and processed with minfi package. Probes with NAs (defined by $P > 0.01$) were discarded. The filtered data was subsequently normalized with BMIQ, resulting in a normalized data matrix for 479076 probes and 68 samples.

Brokaw

The 450k dataset from Brokaw et al. [20] profiled DNAm in middle temporal gyrus (MTG) and the cerebellum (CRB) from controls and AD cases was obtained from the NCBI GEO website under the accession number GSE134379 (https://www.ncbi.nlm.nih.gov/geo/query/acc.cgi?acc=GSE134379). The file "GSE134379_RAW.tar" which contains the IDAT files was downloaded and processed with minfi package. Probes with NAs (defined by $P > 0.01$) were discarded. The filtered data was subsequently normalized with BMIQ, resulting in a normalized data matrix for 370843 probes and 808 samples.

SUPPLEMENTARY REFERENCES

1. Pai S, Li P, Killinger B, Marshall L, Jia P, Liao J, Petronis A, Szabó PE, Labrie V. Differential methylation of enhancer at IGF2 is associated with abnormal dopamine synthesis in major psychosis. Nat Commun. 2019; 10:2046. https://doi.org/10.1038/s41467-019-09786-7 PMID:31053723

2. Gasparoni G, Bultmann S, Lutsik P, Kraus TFJ, Sordon S, Vlcek J, Dietinger V, Steinmaurer M, Haider M, Mulholland CB, Arzberger T, Roeber S, Riemenschneider M, et al. DNA methylation analysis on purified neurons and glia dissects age and Alzheimer's disease-specific changes in the human cortex. Epigenetics Chromatin. 2018; 11:41. https://doi.org/10.1186/s13072-018-0211-3 PMID:30045751

3. Kozlenkov A, Jaffe AE, Timashpolsky A, Apontes P, Rudchenko S, Barbu M, Byne W, Hurd YL, Horvath S, Dracheva S. DNA Methylation Profiling of Human Prefrontal Cortex Neurons in Heroin Users Shows Significant Difference between Genomic Contexts of Hyper- and Hypomethylation and a Younger Epigenetic Age. Genes (Basel). 2017; 8:152. https://doi.org/10.3390/genes8060152 PMID:28556790

4. Guintivano J, Aryee MJ, Kaminsky ZA. A cell epigenotype specific model for the correction of brain cellular heterogeneity bias and its application to age, brain region and major depression. Epigenetics. 2013; 8:290–302. https://doi.org/10.4161/epi.23924 PMID:23426267

5. Hannon E, Dempster EL, Davies JP, Chioza B, Blake GET, Burrage J, Policicchio S, Franklin A, Walker EM, Bamford RA, Schalkwyk LC, Mill J. Quantifying the proportion of different cell types in the human cortex using DNA methylation profiles. BMC Biol. 2024; 22:17. https://doi.org/10.1186/s12915-024-01827-y PMID:38273288

6. de Witte LD, Wang Z, Snijders GLJL, Mendelev N, Liu Q, Sneeboer MAM, Boks MPM, Ge Y, Haghighi F. Contribution of Age, Brain Region, Mood Disorder Pathology, and Interindividual Factors on the Methylome of Human Microglia. Biol Psychiatry. 2022; 91:572–81. https://doi.org/10.1016/j.biopsych.2021.10.020 PMID:35027166

7. Murphy TM, Crawford B, Dempster EL, Hannon E, Burrage J, Turecki G, Kaminsky Z, Mill J. Methylomic profiling of cortex samples from completed suicide cases implicates a role for PSORS1C3 in major depression and suicide. Transl Psychiatry. 2017; 7:e989. https://doi.org/10.1038/tp.2016.249 PMID:28045465

8. Rydbirk R, Folke J, Busato F, Roché E, Chauhan AS, Løkkegaard A, Hejl AM, Bode M, Blaabjerg M, Møller M, Danielsen EH, Brudek T, Pakkenberg B, et al. Epigenetic modulation of AREL1 and increased HLA expression in brains of multiple system atrophy patients. Acta Neuropathol Commun. 2020; 8:29. https://doi.org/10.1186/s40478-020-00908-7 PMID:32151281

9. Torabi Moghadam B, Etemadikhah M, Rajkowska G, Stockmeier C, Grabherr M, Komorowski J, Feuk L, Carlström EL. Analyzing DNA methylation patterns in subjects diagnosed with schizophrenia using machine learning methods. J Psychiatr Res. 2019; 114:41–7. https://doi.org/10.1016/j.jpsychires.2019.04.001 PMID:31022588

10. Xu H, Wang F, Liu Y, Yu Y, Gelernter J, Zhang H. Sex-biased methylome and transcriptome in human prefrontal cortex. Hum Mol Genet. 2014; 23:1260–70. https://doi.org/10.1093/hmg/ddt516 PMID:24163133

11. Huynh JL, Garg P, Thin TH, Yoo S, Dutta R, Trapp BD, Haroutunian V, Zhu J, Donovan MJ, Sharp AJ, Casaccia P. Epigenome-wide differences in pathology-free regions of multiple sclerosis-affected brains. Nat Neurosci. 2014; 17:121–30. https://doi.org/10.1038/nn.3588 PMID:24270187

12. Viana J, Hannon E, Dempster E, Pidsley R, Macdonald R, Knox O, Spiers H, Troakes C, Al-Saraj S, Turecki G, Schalkwyk LC, Mill J. Schizophrenia-associated methylomic variation: molecular signatures of disease and polygenic risk burden across multiple brain regions. Hum Mol Genet. 2017; 26:210–25. https://doi.org/10.1093/hmg/ddw373 PMID:28011714

13. Markunas CA, Semick SA, Quach BC, Tao R, Deep-Soboslay A, Carnes MU, Bierut LJ, Hyde TM, Kleinman JE, Johnson EO, Jaffe AE, Hancock DB. Genome-wide DNA methylation differences in nucleus accumbens of smokers vs. nonsmokers. Neuropsychopharmacology. 2021; 46:554–60. https://doi.org/10.1038/s41386-020-0782-0 PMID:32731254

14. Policicchio S, Washer S, Viana J, Iatrou A, Burrage J, Hannon E, Turecki G, Kaminsky Z, Mill J, Dempster EL, Murphy TM. Genome-wide DNA methylation meta-analysis in the brains of suicide completers. Transl Psychiatry. 2020; 10:69. https://doi.org/10.1038/s41398-020-0752-7 PMID:32075955

15. Semick SA, Bharadwaj RA, Collado-Torres L, Tao R, Shin JH, Deep-Soboslay A, Weiss JR, Weinberger DR, Hyde TM, Kleinman JE, Jaffe AE, Mattay VS. Integrated DNA methylation and gene expression profiling across multiple brain regions implicate novel genes in Alzheimer's disease. Acta Neuropathol. 2019; 137:557–69. https://doi.org/10.1007/s00401-019-01966-5 PMID:30712078

16. Pidsley R, Y Wong CC, Volta M, Lunnon K, Mill J, Schalkwyk LC. A data-driven approach to preprocessing Illumina 450K methylation array data. BMC Genomics. 2013; 14:293. https://doi.org/10.1186/1471-2164-14-293 PMID:23631413

17. Smith RG, Hannon E, De Jager PL, Chibnik L, Lott SJ, Condliffe D, Smith AR, Haroutunian V, Troakes C, Al-Sarraj S, Bennett DA, Powell J, Lovestone S, et al. Elevated DNA methylation across a 48-kb region spanning the HOXA gene cluster is associated with Alzheimer's disease neuropathology. Alzheimers Dement. 2018; 14:1580–8. https://doi.org/10.1016/j.jalz.2018.01.017 PMID:29550519

18. Horvath S, Langfelder P, Kwak S, Aaronson J, Rosinski J, Vogt TF, Eszes M, Faull RL, Curtis MA, Waldvogel HJ, Choi OW, Tung S, Vinters HV, et al. Huntington's disease accelerates epigenetic aging of human brain and disrupts DNA methylation levels. Aging (Albany NY). 2016; 8:1485–512. https://doi.org/10.18632/aging.101005 PMID:27479945

19. Watson CT, Roussos P, Garg P, Ho DJ, Azam N, Katsel PL, Haroutunian V, Sharp AJ. Genome-wide DNA methylation profiling in the superior temporal gyrus reveals epigenetic signatures associated with Alzheimer's disease. Genome Med. 2016; 8:5. https://doi.org/10.1186/s13073-015-0258-8 PMID:26803900

20. Brokaw DL, Piras IS, Mastroeni D, Weisenberger DJ, Nolz J, Delvaux E, Serrano GE, Beach TG, Huentelman MJ, Coleman PD. Cell death and survival pathways in Alzheimer's disease: an integrative hypothesis testing approach utilizing -omic data sets. Neurobiol Aging. 2020; 95:15–25. https://doi.org/10.1016/j.neurobiolaging.2020.06.022 PMID:32745806
