# Supplementary material for: Cell-type specific epigenetic clocks to quantify biological age at cell-type resolution
Source: Aging (Albany NY). 2024 Dec 29;16(22):13452–504. doi: 10.18632/aging.206184 (PMC11723652; doi:10.18632/aging.206184)
Supplement: Supplementary Tables 8-11 [file aging-16-206184-s004.pdf]

SUPPLEMENTARY TABLES

Supplementary Table 8. The optimal Elastic Net predictor representing the hepatocyte-specific clock.

Probe	β	Gene symbol	NCBI ID
cg02918910	−6.017436881	MLLT11	10962
cg05675373	−5.459241272	KCNC4	3749
cg06085726	−2.176126012	PLEKHG5	57449
cg06850099	−1.163595542	LAMC1	3915
cg10483534	−11.27967918	DISC2	27184
cg15458155	−2.432514762	ODF2L	57489
cg26883161	0.549543803	RGL1	23179
cg21211367	34.7997767		
cg24996440	0.556654931		
cg03553587	5.580119066		
cg04461197	−7.79656029	SPATA16	83893
cg10719144	−7.169039318		
cg11320271	1.542575768	CCDC36	339834
cg14098708	15.73487273	SLC6A6	6533
cg16978797	0.359053738	ARHGEF3	50650
cg11956467	6.515843914	SORBS2	8470
cg04670168	19.46881394	MYOZ3	91977
cg08271852	1.41841243		
cg09457131	0.032538949		
cg11801489	3.521123817		
cg14129473	−0.357563612	DAP	1611
cg16016281	3.376959884		
cg26919818	21.54003737	N4BP3	23138
cg05441830	−7.915169358		
cg13705956	−2.34826709	SMOC2	64094
cg14111380	−1.25046115	GABBR1	2550
cg09803262	−3.796130414	DLX6AS	285987
cg10542223	13.85943901	ZNF815	401303
cg10741603	4.520953706	SDK1	221935
cg14646653	0.223822725	GLI3	2737
cg21329649	−0.084885825	MAD1L1	8379
cg25644224	5.864462497	PTPRN2	5799
cg26836793	−2.691338723	RP9P	441212
cg00935717	−0.150122949	ZMAT4	79698
cg07350076	4.765699061	FAM66A	100133172
cg03020810	0.76432236	TLX1	3195
cg05719612	9.687510549	CACNB2	783
cg11478495	5.014419362	PRKG1	5592
cg14824052	2.470626826		
cg20475550	−0.230176091	PPP1R3C	5507
cg20530855	−2.132530095		

cg24202448	9.199788218		
cg24353740	36.27907046	KCNK18	338567
cg09036621	−4.604553593	PRDM10	56980
cg11782387	−4.345922791		
cg22204050	−6.92582377	FAM90A1	55138
cg24418063	−2.75996521	ATN1	1822
cg06170065	−1.035475913		
cg08112616	25.35109122	MEG8	79104
cg13222081	2.22617245	MRPL52	122704
cg02038233	−6.204072378	DUOX1	53905
cg02736313	−45.91000831	MEGF11	84465
cg09207718	40.84797516	CYP1A2	1544
cg22242148	8.916645472		
cg22492020	0.000210105	TIPIN	54962
cg23203997	−0.425526553		
cg24799460	8.864890338	TARSL2	123283
cg27057329	1.793435599		
cg14696348	−1.63049215	MC1R	4157
cg00735218	−2.253242355	DNAH17	8632
cg19909658	1.643780317	CDC6	990
cg26749306	−3.784572463	METRNL	284207
cg17192381	10.38975753	BCL2	596
cg02412123	−20.87621835	SIGLEC9	27180
cg07517893	28.27230223	PPP1R13L	10848
cg21940568	3.440818072	NCRNA00085	147650
cg00633740	−4.469064195	EDN3	1908
cg07474022	4.649140961	SRC	6714
cg15333674	5.226801949		
cg18170680	1.471564905		

Columns label the probeID, the estimated regression coefficient, gene symbol and NCBI Identifier.

Supplementary Table 9. The optimal Elastic Net predictor representing the liver-clock.

Probe	β	Gene symbol	NCBI ID
cg00147160	0.066771418	CNKSR1	10256
cg04055049	−0.469147506	GPR153	387509
cg07014742	8.956233592	DEDD	9191
cg08639523	−7.425961333	HIST3H2BB	128312
cg09084829	−0.379237029	KCNH1	3756
cg10501085	−0.967451996	SLC30A2	7780
cg11037787	−0.216347627	PLA2G2A	5320
cg11837451	−0.666694294	KCTD3	51133
cg19860717	−0.063381391	PTPN14	5784
cg22792674	−11.50674235	VPS72	6944
cg27552960	−4.447482513	ARF1	375

cg01138171	−0.779451213	ARHGEF4	50649
cg03602297	1.741921083	ALK	238
cg03956823	3.411298693	CHRND	1144
cg11796910	1.9510978	ROCK2	9475
cg12476443	4.327233425	C2orf39	92749
cg23059725	−7.400779735	WDR35	57539
cg26469081	−0.847409586		
cg00480331	1.458519337	BCL6	604
cg01631215	−2.943168366	SUCLG2	8801
cg17631042	−0.044251663	GOLGA4	2803
cg20668838	−2.712245058	PCCB	5096
cg24572577	−3.016548007	SLC41A3	54946
cg02647878	1.045446946	RNF175	285533
cg15848095	0.705701448		
cg27650726	−5.014070673	MRFAP1L2	93622
cg04671082	2.063865623		
cg16049600	0.187949221	PCDHB11	56125
cg01015199	1.635787049	GCLC	2729
cg16866653	51.19827934	BRP44L	105216101
cg22735834	17.66787787	PPP1R14C	81706
cg23528708	−1.501442393	CCDC90A	63933
cg25589039	0.41920236	C6orf70	55780
cg02190873	−3.651450381	PDIA4	9601
cg04727332	−0.698193261	IGFBP3	3486
cg04841607	2.510801346	C7orf33	202865
cg07023501	−0.382460827		
cg08097290	14.04520054	MGC87042	256227
cg09642951	28.95375853	EPHA1	2041
cg10548292	1.556617492		
cg21577626	−1.052262676	EPHB6	2051
cg02396865	1.267908237	DSCC1	79075
cg02605601	−0.127802997		
cg03069902	3.330232605	C10orf12	84458
cg15322963	−5.642289186	IDE	3416
cg22576165	5.496227518		
cg24402267	10.10386528		
cg07200877	−0.391044839	SPRYD5	84767
cg12139707	−2.247439968	ATM	472
cg13654391	0.27780663	DKFZp779M0652	374387
cg15248242	1.967578063		
cg23030863	−4.468072544	JAM3	83700
cg24742649	−1.315776892	OR51G1	79324
cg14120112	−1.819998161	VWF	7450
cg19535609	4.742022158	LRRC43	254050
cg22221074	−0.373301431	EFCAB4B	84766

cg27632050	4.193695173	RASA3	22821
cg04963651	−3.046651002	ERH	2079
cg22831425	31.60062472		
cg25487438	−1.253946288	DCAF11	80344
cg06486467	0.128498105		
cg18548762	−10.21521945		
cg19642394	−4.217757262	UBL7	84993
cg20901246	1.293243827		
cg22929280	−0.856017399	MIR549	69313
cg02741291	0.666219074	UBN1	29855
cg05304184	−3.141257729		
cg09285525	8.136402387	SLC7A5	8140
cg10150962	−2.324888102		
cg27106356	−1.512703347	TNP2	7142
cg01574134	−1.648422529	MIR636	693221
cg04124526	−0.360735134	FAM171A2	284069
cg06144469	7.583048847	OTOP3	347741
cg07142886	6.768928912		
cg13868393	−1.535866415	SEC14L1	6397
cg15138339	−0.07961536	COASY	80347
cg22352029	1.924760193	PYY	5697
cg02423030	−0.805027326	MAST1	22983
cg06456376	8.581426017		
cg07543967	8.03627842	SULT2B1	6820
cg10604333	0.332748993	ZNF222	7673
cg13551088	−0.045109103	ZNF573	126231
cg15429854	−7.110702776	SLC27A1	376497
cg20118823	0.400570278	TCF3	6929
cg11399508	−0.704090071	DSCR9	257203
cg01133262	−4.790660983	KDELR3	11015
cg01585852	6.523374051	MIF	4282
cg07917473	−12.28794972		
cg10648908	−2.631696356	ARFGAP3	26286
cg15508776	0.276760274	CACNA1I	8911

Columns label the probeID, the estimated regression coefficient, gene symbol and NCBI identifier.

Supplementary Table 10. The overlap between brain cell-type specific (CTS) clocks and DamAge clock.

Brain CTS clock	Brain CTS clock coef	DamAge coef	Gene	Regulatory region
Neu-In	−0.004419393	0.670384139	SESN2	TSS1500
Neu-In	−0.138237554	0.469640644	SGMS1	5′UTR
Neu-In	−0.386904875	0.436183395	EPS8L2	5′UTR
Neu-In	0.083751079	0.501032631		
Neu-Sin	−0.001363156	0.670384139	SESN2	TSS1500
Neu-Sin	0.081413726	0.239157373	ZNF642	TSS1500

Neu-Sin	−3.016294236	0.469640644	SGMS1	5′UTR
Neu-Sin	−15.09060534	0.436183395	EPS8L2	5′UTR
Neu-Sin	9.088271948	0.501032631		
Glia-In	0.132534035	0.203634862		
Glia-In	−0.153640725	0.716232962		
Glia-In	−0.019645819	0.794712929	ST3GAL4	1stExon; 5′UTR
Glia-In	−0.061458766	−13.49218833	CBX7	Body
Glia-Sin	2.821787117	0.203634862		
Glia-Sin	−6.85227349	0.716232962		
Glia-Sin	0.001254957	0.390334572	TOMM40L	TSS1500
Glia-Sin	−2.467746175	0.794712929	ST3GAL4	1stExon; 5′UTR
Glia-Sin	−4.937783763	−13.49218833	CBX7	Body

Columns label the probeID, the brain CTS clock, the brain CTS clock estimated regression coefficient, the DamAge clock estimated regression coefficient, the mapped gene, the mapped regulatory region.

Supplementary Table 11. Two Neu-In clock CpGs that define cis-mQTLs with SNPs that have been associated with Alzheimer's Disease (AD).

CpG	SNP	PvalInADgwas	Chromosome	SNP_location	CpG_location	CpGmappedGene	mQTLtype
cg24519157	rs6024860	1.21E-09	20	56408730	56412605	CASS4	cis
cg24519157	rs1884913	3.55E-10	20	56409008	56412605	CASS4	cis
